# Deamidation Promotes AGE-Modifications in Human Lens γS-Crystallin

**DOI:** 10.1021/acs.biochem.6c00087

**Published:** 2026-04-07

**Authors:** Sudipta Panja, Ram H. Nagaraj

**Affiliations:** Department of Ophthalmology, University of Colorado Anschutz, Aurora, Colorado 80045, United States

## Abstract

Deamidation and advanced glycation end products (AGEs) are among the major post-translational modifications (PTMs) in eye lens proteins. Due to deamidation and AGE formation, proteins may aggregate and scatter light, contributing to lens aging and cataract formation. So far, the relation between the two PTMs remains poorly understood. γS-crystallin (γSC), a major subtype of γ-crystallin, undergoes significant modifications through deamidation, especially at the surface-exposed asparagine residues, N14, N76, and N143. In this study, deamidation of γSC was mimicked by mutating asparagine residues to aspartic acid residues. The deamidation mimics were then incubated with a glycating mixture, and AGE formation in proteins was evaluated by LC–MS/MS. Results indicate that deamidation promotes the formation of both non-cross-linking (CML or CEL) and cross-linking AGEs (GOLD, MOLD, or pentosidine) in lysine residues. AGE formation in arginine residues (e.g., MG-H3) is mostly unaffected. Comparative analysis shows that N14D, N143D, and N14DN76DN143D (triple deamidated, TD) consistently accumulated more AGEs than native γSC. Oxidation with 2 mM GSSG led to increased disulfide-linked cross-linking in deamidated γSC. Upon glycation, the deamidated and oxidized γSC accumulated more AGEs than deamidated γSC; however, the specific AGE levels followed the same trend as deamidated γSC. The results suggest that deamidation promotes AGE formation in γSC, and further oxidation makes it even more susceptible to AGE modifications. The combined interdependent effects of deamidation, oxidation, and AGE modifications could therefore contribute to protein cross-linking and aggregation during lens aging and cataract formation.

## Graphical abstract

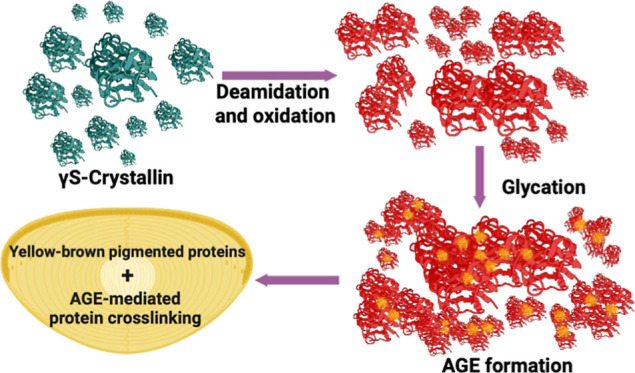

## Introduction

Post-translational modifications (PTMs) are major contributors to lens aging.^1–3^ Since lens proteins have minimal turnover, PTMs such as racemization and deamidation,^4–6^ glycation,^7^ oxidation,^8–10^ proteolytic degradation and truncation,^11,12^ dehydroalanine formation^13^ and transglutaminase-mediated cross-linking^14,15^ accumulate with lens aging. Among these modifications, deamidation is prominent.^5,6^

Deamidation occurs in asparagine and glutamine residues in lens crystallins (Figure 1A). In the case of asparagine, isoaspartic acid and aspartic acid are the major products of deamidation^16^ and in the case of glutamine, glutamic acid is the major product.^17^ Deamidated crystallins have been linked to lens aging and cataract formation.^16^ α-Crystallin in cataract lenses exhibits a higher degree of deamidation compared to that in normal lenses.^18^ The impact of age-related deamidation on the structure and function of α-crystallin was examined through various mutants that mimic deamidation, including αA-crystallin (αAC) mutants (N101D, N123D, and N101DN123D) and αB-crystallin (αBC) mutants (N78D, N146D, and N78DN146D).^19^ These αAC mutants showed changes in secondary structure and local environment; notably, deamidation at N123 resulted in a marked reduction in chaperone activity.^20^ Additionally, deamidation at N146 influenced both the structural integrity and functional capacity of αBC.^21^ The most prevalent in vivo deamidation sites have been observed in β/γ-crystallins, and they occur in the solvent-exposed regions of proteins. Deamidation not only changes the structure of these crystallins but also increases their susceptibility to aggregation. For example, deamidation of βA3C and βB2C increases their susceptibility to aggregation.^16,22^ Furthermore, deamidation of specific residues in crystallins is higher in aggregated water-insoluble proteins than in less aggregated water-soluble proteins,^3^ reiterating that deamidation contributes to protein aggregation in the lens.

**Figure 1:**
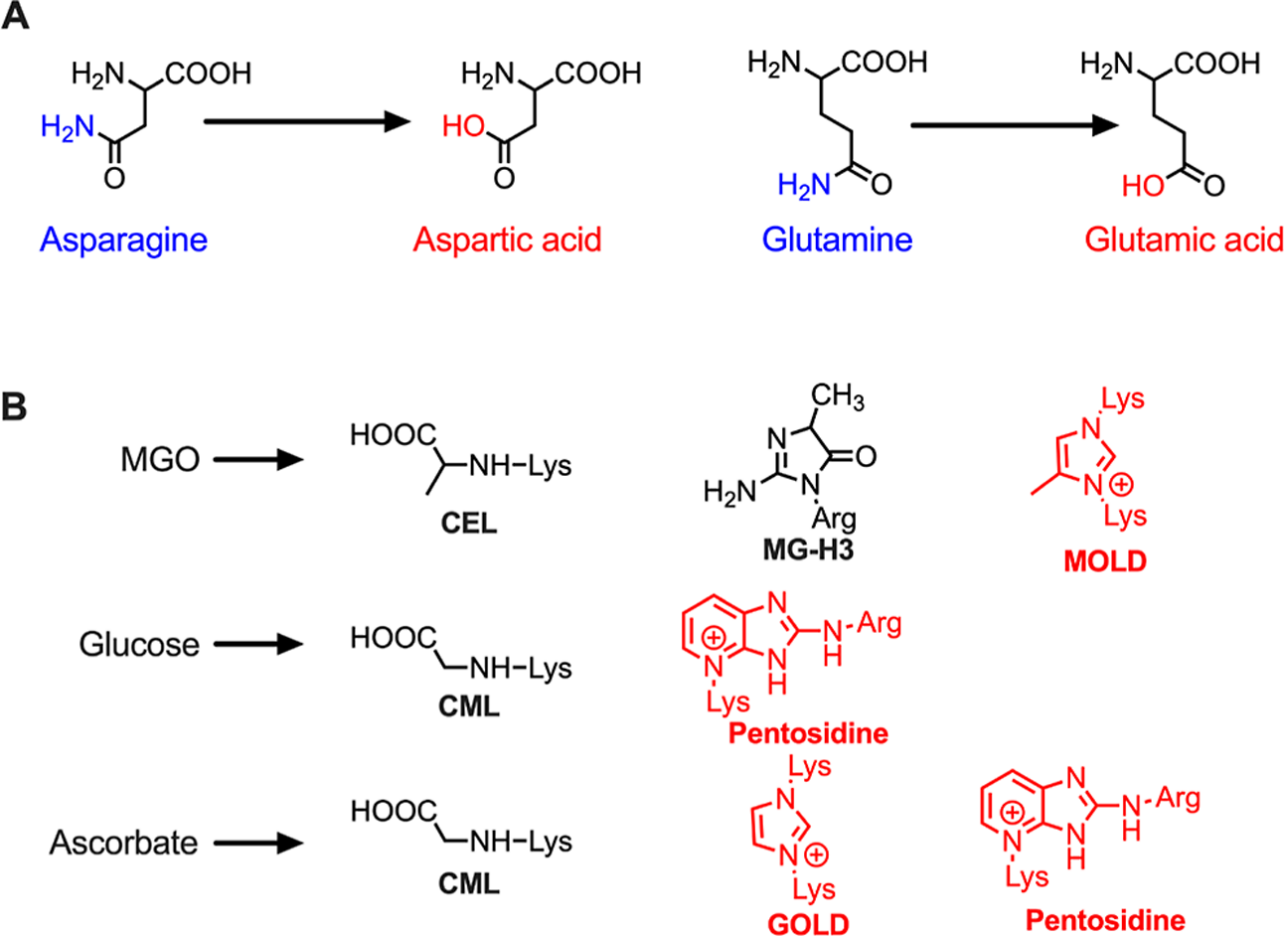
Deamidation of asparagine and glutamine residues in proteins (A). The structure of AGEs from MGO, d-glucose, and ascorbate is shown (B). AGEs shown were measured in this study using LC–MS/MS. Cross-linking AGEs are shown in red.

GammaS-crystallin (γSC) is a major γ-crystallin subtype in the human lens. Deamidation in γSC has been well documented at N14, N76, and N143.^17^ Several studies have shown that introducing aspartic acid residues at these asparagine residues increases γSC’s propensity to destabilize and aggregate due to lowering of the unfolding temperature and destabilization of the β-sheet packing.^5,23–26^ Together, these observations provide strong evidence that deamidation contributes to protein modification and aggregation during lens aging.

Another significant age-related PTM in lens proteins is the formation of advanced glycation end products (AGEs).^27,28^ AGEs form through nonenzymatic reactions between the carbonyl groups of sugars and carbonyl compounds and the amino groups of lysine and arginine residues. In vivo, various sugars, such as glucose, fructose, and ribose, as well as oxidation products of ascorbate, contribute to AGE formation.^29,30^ Alpha-dicarbonyl compounds such as glyoxal (GO) and methylglyoxal (MGO), originating from metabolic reactions, sugar breakdown, Maillard reaction intermediates, ascorbate oxidation, and lipid peroxidation, are highly reactive AGE precursors.^31^ AGEs are generally classified into two categories: non-cross-linking and cross-linking. Non-cross-linking AGEs include *N* ^ε^-carboxymethyllysine (CML) and *N* ^ε^-carboxyethyllysine (CEL), which form on lysine residues, as well as methylglyoxal-derived hydroimidazolone (MG-H3) and argpyrimidine, which form on arginine residues. Cross-linking AGEs include pentosidine, glyoxal-lysine dimer (GOLD), and methylglyoxal-lysine dimer (MOLD), which are formed as lysine–lysine cross-links (Figure 1B). Additional cross-linking AGEs, such as GODIC and MODIC are formed between lysine and arginine residues in proteins.^32^ These cross-linking AGEs are likely to promote the formation of high-molecular-weight (HMW) proteins, a hallmark of aging in the human lens that contributes to protein insolubilization and aggregation.^33,34^

The lens contains high levels of reduced glutathione (GSH), which protects against oxidative damage and inhibits ascorbate oxidation and AGE formation.^35^ GSH levels and antioxidant enzyme activities decrease with age, leading to increased oxidative stress and glycation damage.^35,36^ Studies have shown that AGEs accumulate steadily in human lenses with age and are elevated in cataractous lenses.^37^ Our previous work demonstrated a strong link between lens stiffness and AGE buildup, indicating a role for AGEs in the development of presbyopia.^32^ Furthermore, we found that carboxitin, a chimeric compound combining mercaptoethylguanidine and GSH diethyl ester, which supplies GSH and traps α-dicarbonyl AGE precursors, inhibits AGE formation and reduces lens stiffness, further supporting the role of AGEs in lens aging and presbyopia.^38^

Deamidation and AGE formation in proteins occur simultaneously in aging lenses. Several questions remain unanswered about the relationship between these two PTMs. For example, how does deamidation affect AGE formation? Does deamidation promote protein cross-linking through AGE formation? To date, no reports have investigated the effect of deamidation on AGE formation. Protein oxidation is another PTM that forms disulfide bonds, thereby altering protein folding and potentially promoting aggregation. Since both deamidation and oxidation can alter protein structure, they might also expose more lysine and arginine residues for AGE modification. In this study, we investigated how prior deamidation and oxidation influence AGE modifications in human γSC. We used γSC as a representative molecule, so the findings could also apply to other lens proteins and to proteins in tissues that show high levels of AGEs.

## Experimental Procedures

### Reagents

d-Glucose (Cat# G7528), methylglyoxal (MGO, Cat# M0252), l-ascorbate sodium salt (Cat# A7631), and GSH (Cat# G6529) were purchased from Sigma-Aldrich (St. Louis, MO). GSSG (Cat# 151193) was purchased from MP Biomedicals (Irvine, CA). All other chemicals were analytical grade.

### Cloning, Expression, and Purification of γSC and Its Mutants

Genes encoding each protein were cloned, expressed in *Escherichia coli* and purified, as previously described.^39^ Briefly, genes encoding wild-type (WT) and deamidated γSC were synthesized by Twist Bioscience and inserted into a pET-SUMO expression vector using BamH1 and Xho1 sites. After cloning, the genes were transformed into *E. coli* BL21 (DE3) cells. Plasmids were isolated from the transformed cells with a Qiagen plasmid miniprep kit, and the sequences were confirmed. Bacterial constructs encoding the proteins were grown in Lysogeny Broth media at 37 °C. Protein expression was induced with 0.5 mM isopropyl-β-d-thiogalactopyranoside at an absorbance of 0.6–0.8 at 600 nm. Protein purification was performed using a Ni-Sepharose affinity column from Cytiva (Marlborough, MA). The N-terminal polyhistidine-SUMO tag was removed by treatment with Ulp1 protease. Finally, purified proteins were dialyzed against 100 mM phosphate buffer, pH 7.4 and protein homogeneity was verified by SDS-PAGE.

### Circular Dichroism (CD) Fluorescence Experiments

Far- and near-UV CD spectra were recorded in a Chirascan Plus spectophotometer (Applied Photophysics, UK) at 25 °C. Far-UV CD spectra were recorded in a 0.1 cm path-length quartz cell at a protein concentration of 0.25 mg/mL, with a scanning speed of 1 s per wavelength and a 1 nm bandwidth. Near-UV CD spectra were recorded in a 1 cm path-length quartz cell at a protein concentration of 1 mg/mL, a scanning speed of 1 s per wavelength, and a 1 nm bandwidth. Final spectra were acquired after averaging three successive measurements.

Fluorescence spectra were measured using a Fluoromax-4 spectrofluorometer (Horiba Jobin Yvon, Edison, NJ). Tryptophan fluorescence was recorded at an excitation wavelength of 295 nm; the spectra were recorded from 310 to 470 nm at 2 nm intervals with a 5 nm slit width.

### Incubation of γSC with AGE Precursors

Human recombinant native γSC and deamidated variants (2 mg/mL) were incubated for 5 days at 37 °C under sterile conditions with 25 mM d-glucose, 2 mM sodium ascorbate, and 250 μM MGO in 100 mM sodium phosphate buffer, pH 7.4. After incubation, the proteins were dialyzed against PBS for 16 h, at 4 °C.

### Measurement of AGEs by LC–MS/MS

Samples were acid hydrolyzed and dried using a previously established protocol.^40^ The digested samples were then analyzed for AGEs by LC–MS/MS method with standard addition, as previously described.^40^ The AGE levels were expressed as nmol/mg protein.

### Measurement of Pentosidine

Proteins were acid-hydrolyzed as above. The acid-hydrolyzed samples were analyzed for pentosidine by UPLC (column: Waters Acquity HSS T3 1.8 μM 2.1 × 100 mm) with a fluorescence detector, as described previously.^32^ Briefly, a linear gradient program was used with solvent A: 100% water with 0.12% heptafluorobutyric acid, and solvent B: 80% acetonitrile with 0.12% heptafluorobutyric acid. The program was as follows: 0–5 min: 10% B; 5–7 min: 30% B; 7–7.5 min: 70% B; 7.5–10 min: 100% B; and 10–12.9 min: 10% B, at a flow rate of 0.5 mL/min. The eluted sample was monitored for the fluorescence signal of pentosidine (excitation/emission wavelengths of 335/385 nm) with an inline fluorescence detector. Pentosidine content in samples was calculated from a standard curve generated with synthetic pentosidine.

### Protein-Thiol Estimation

Ten micrograms of dialyzed WT-γSC and deamidated variants were used to estimate thiol content using the Thiol Quantification Assay Kit (Abcam, Cambridge, MA), with reduced GSH as the standard.

### Surface Exposed Lysine Residues

WT-γSC and deamidated mutant variants (200 μg/0.3 mL) were incubated with 0.15 mL of 0.01% TNBS in 0.1 M sodium bicarbonate buffer, pH 8.5, at RT for 2 h. After incubation, 0.15 mL of 10% SDS was added, and the reaction was neutralized by adding 0.075 mL of 1 M HCl. The absorbance was measured at 420 nm.

### Statistics

The data are presented as the mean ± standard deviation (SD). Groups were compared using one-way ANOVA multiple comparsion tests (Graphpad Prism, Version 10).

## Results

### Purification and Characterization of γSC

Native γSC and deamidated mimics (hereafter referred to as deamidated proteins) were purified, and their purity was verified by SDS-PAGE under reducing conditions (Figure 2A). A single predominant band ∼20 kDa confirmed the high purity of the native γSC and deamidated proteins. Far- and near-UV CD spectra^41^ verified the secondary and tertiary structures of proteins (Figure 2B and C). The far UV-CD spectrum of native γSC indicated a mainly β-sheet structure. No spectral changes were observed after deamidation in any of the proteins. In near-UV-CD, a slight conformational shift was observed following deamidation. However, all deamidated proteins displayed similar structures.

**Figure 2:**
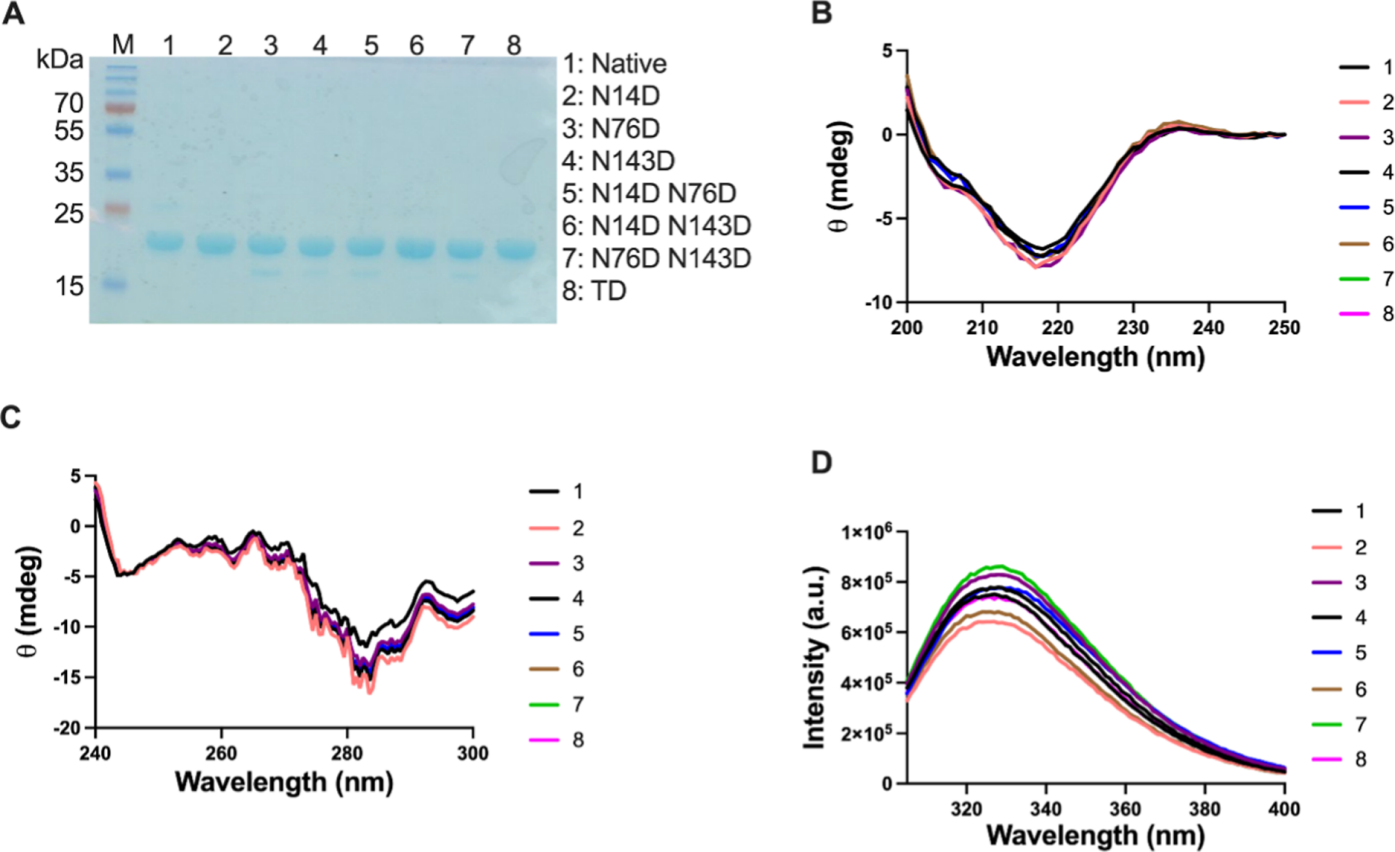
Purity and secondary structure characterization of native and deamidated γSC. Native γSC and its deamidated forms showed a single band on SDS-PAGE at ∼20 kDa (A). Far-UV-CD showed no structural changes after deamidation (B), whereas near-UV-CD showed minimal change (C). Tryptophan fluorescence showed a slight variation in tertiary structure after deamidation (D). M = molecular weight markers.

The native and the deamidated γSCs contain four tryptophan residues and 14 tyrosine residues. The emission spectrum of the protein after selective excitation for tryptophan at 295 nm showed a fluorescence emission maximum at 330 nm (Figure 2D). A slight variation in fluorescence intensity among deamidated proteins indicated a change in micropolarity around tryptophan. The emission maximum after deamidation shifted to red, indicating increased solvent exposure of the tryptophan residues (Figure S1).

### Deamidation Promotes AGE Formation in γSC

The CML levels were significantly higher in single deamidated N14D (146.2%, *p* < 0.0001), N76D (119.1%, *p* < 0.0001), N143D (193.6%, *p* < 0.0001), and TD protein (136.2%, *p* < 0.0001) compared with native nondeamidated γSC (Figure 3A). The double deamidated proteins N14DN143D (212.1% higher, *p* < 0.0001) and N76DN143D (39.9% higher, *p* < 0.05) showed significantly higher levels than native γSC. The levels were 25.5% higher in N14DN76D than in native γSC (Figure S2). Considering all deamidated proteins, CML formation appeared to proceed in the order N14DN143D > N143D > N14D > TD. The CEL levels were also significantly higher in N143D (67.9%, *p* < 0.0001) and TD (30.5%, *p* < 0.001). The double deamidated proteins N14DN143D and N76DN143D showed 26.1% (*p* < 0.01) and 29.1% (*p* < 0.001) higher levels than native γSC, but the levels remained unchanged in N14D (Figure 3B) and N14DN76D (Figure S2). Interestingly, the levels were slightly lower in N76D (43.0%). When all deamidated proteins were considered, CEL formation seemed to follow the order N143D > TD > N76DN143D > N14DN143D. Interestingly, in the arginine-based modification MG-H3, all deamidation variants showed no significant change (Figure 3C), except for N14DN143D (119.0% higher, *p* < 0.0001) (Figure S2).

**Figure 3:**
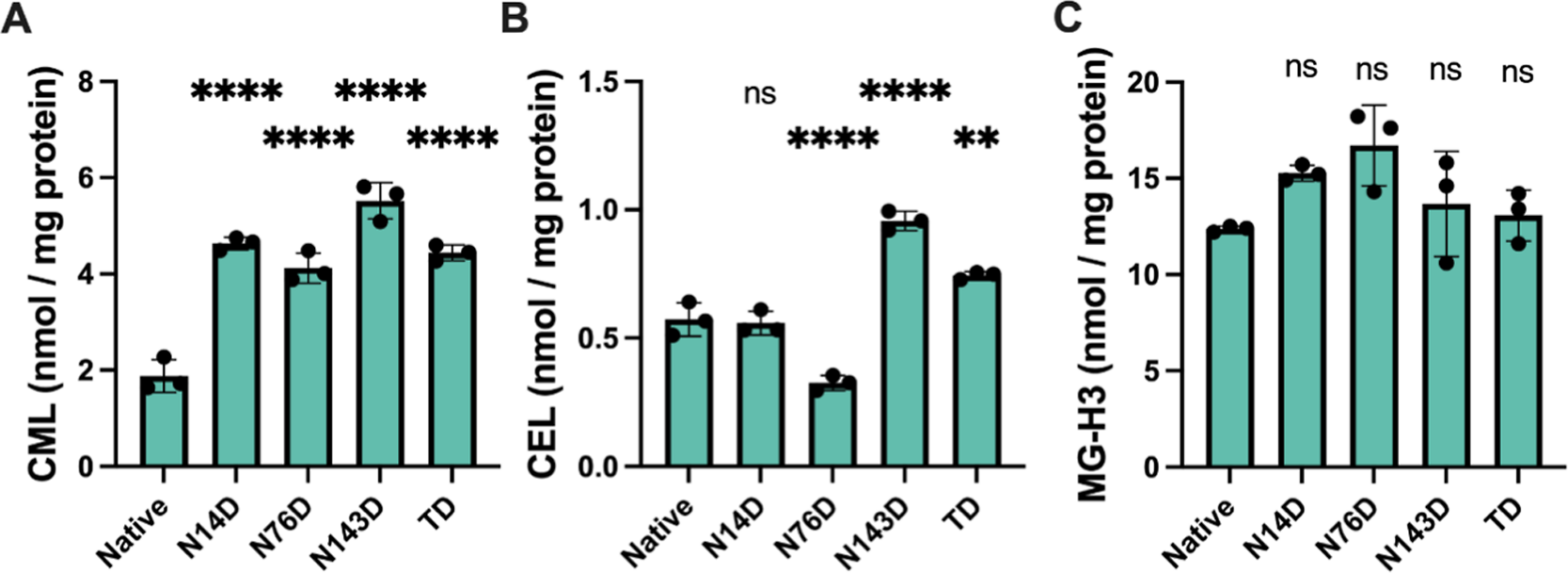
Deamidation enhances the formation of non-cross-linking AGEs in γSC. Native and deamidated γSC were incubated with 25 mM d-glucose, 2 mM ascorbic acid, and 250 μM MGO for 5 days at 37 °C. The proteins were dialyzed, acid hydrolyzed, and analyzed for CML (A) and CEL (B) MG-H3 (C) by LC–MS/MS. The bar graphs represent the mean ± SD of three independent experiments. **p < 0.01, ****p < 0.0001, ns = not significant.

The cross-linking AGE levels were higher in deamidated proteins compared to native γSC (Figures 4 and S3). The increase in GOLD levels with N14D was 64.7% (*p* < 0.01), with N76D it was 98.8% (*p* < 0.001), and with N143D it was 49.4% (*p* < 0.05). The TD had the highest levels at 111.7% (*p* < 0.001) (Figure 4A). The double deamidated N14DN143D also showed significantly higher levels at 57.6% (*p* < 0.01) (Figure S3). The GOLD levels, in order from highest to lowest, were: TD > N76D > N14D > N14DN143D. The MOLD levels were significantly higher in N14D (171.9%, *p* < 0.0001), N143D (106.8%, *p* < 0.001), and TD (78.6%, *p* < 0.05) (Figure 4B). The levels in the double mutant N14DN143D were also significantly higher (99.1%, *p* < 0.0001) (Figure S3). The MOLD levels decreased in the order of N14D > N143D > N14DN143D > N76D > TD. Pentosidine levels were highest in N14DN143D (223.0%, *p* < 0.0001), followed by N14D (176.5%, *p* < 0.0001) and TD (159.4%, *p* < 0.001) (Figures 4C and S3). Overall, N14D and N143D appear to promote cross-linking AGE formation in γSC, similar to what was observed with CML and CEL.

**Figure 4:**
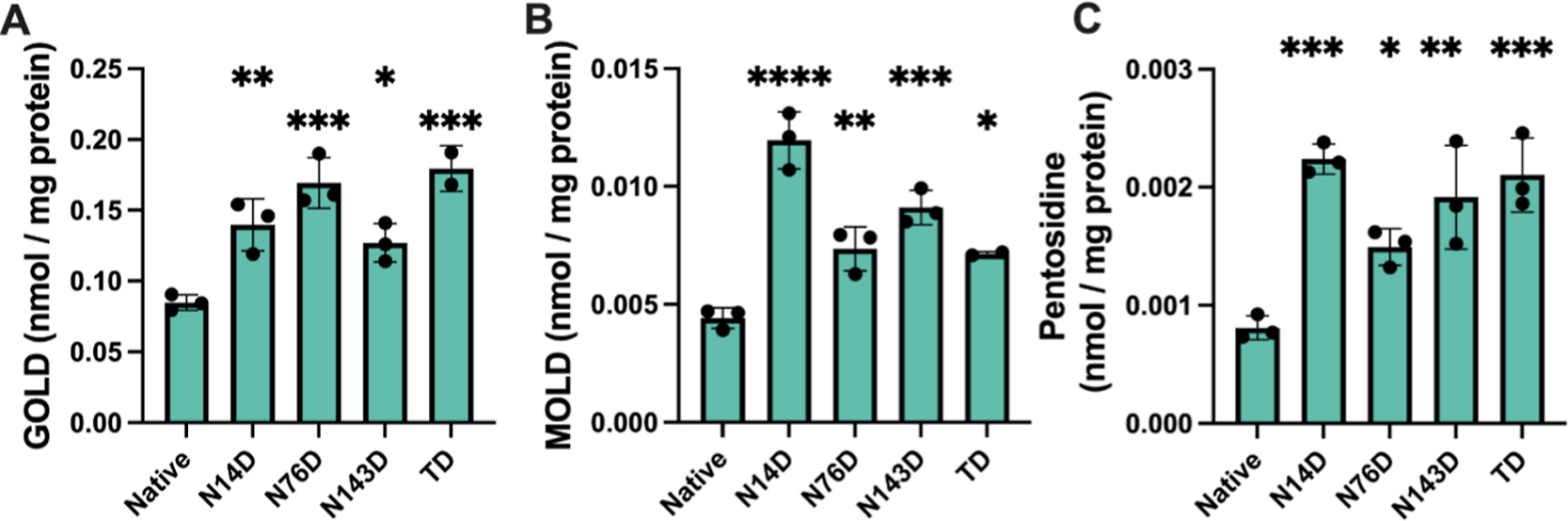
Deamidation accelerates the formation of cross-linking AGEs in γSC. Native and deamidated γSC were glycated and processed as in Figure 1, and subjected to GOLD (A) and MOLD (B) and pentosidine (C) analysis by LC–MS/MS. The bar graphs represent the mean ± SD of three independent experiments. *p < 0.05, **p < 0.01, ***p < 0.001, ****p < 0.0001.

We next investigated whether deamidation exposes additional lysine residues that promote AGE formation. As an example, we investigated the relationship between surface-exposed lysine residues and CML formation. As shown in Figure S4, deamidation increased the number of surface-exposed lysine residues. However, this increase did not correlate with CML levels.

### Oxidized Glutathione Enhances Disulfide Cross-Linking in Deamidated γSC

After oxidation with GSSG (2 mM) for 2 days, free thiol levels were measured. The remaining free thiol was negligible, and all proteins were fully oxidized (Figure 5A). Nonreducing SDS-PAGE was used to assess the formation of HMW species of those proteins after oxidation (Figure 5B). The results (Figure 5C) showed that, except for N14DN76D, all deamidated proteins exhibited HMW species. The results provided clear evidence of increased intermolecular disulfide cross-linking due to oxidation of deamidated proteins.

**Figure 5:**
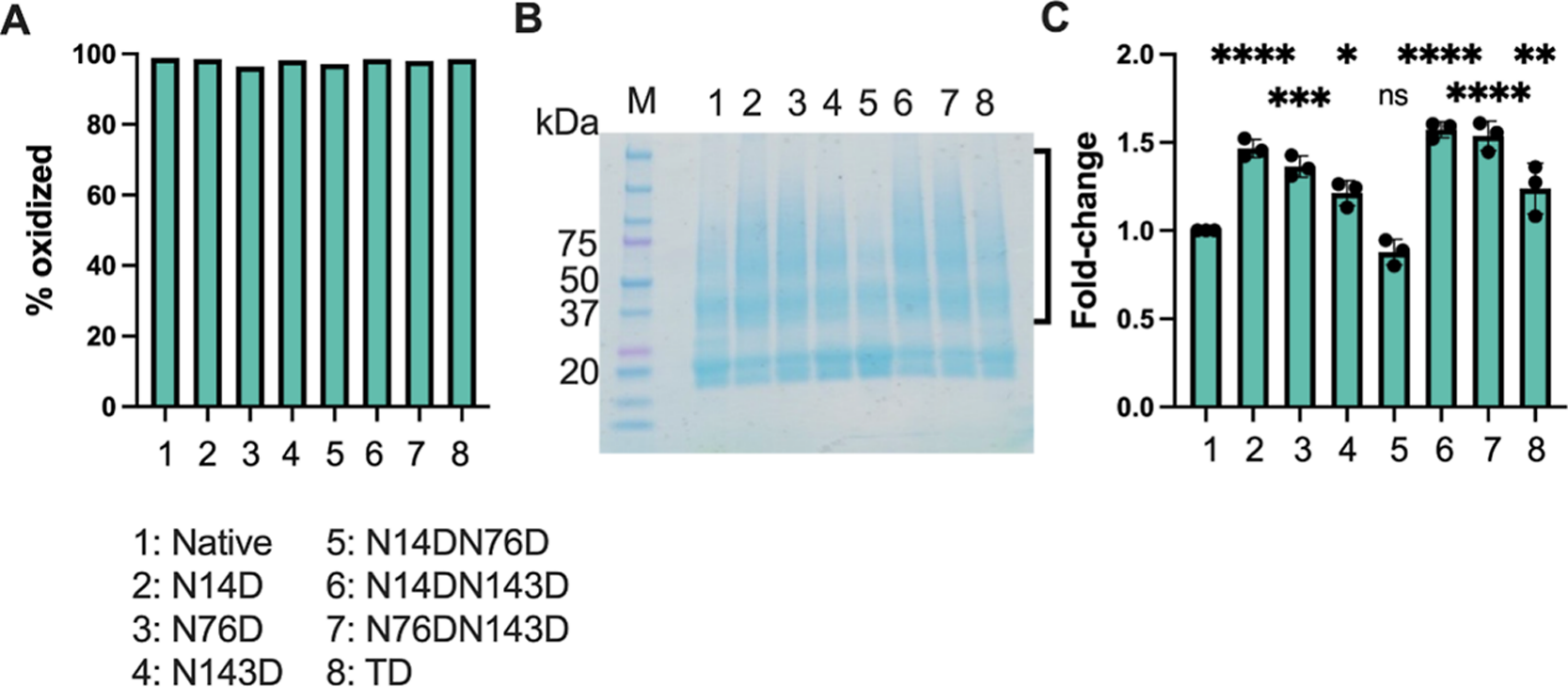
Oxidized and deamidated γSC shows increased cross-linking. γSC was incubated with 2 mM GSSG for 2 days at 37 °C. After dialysis, the protein-free thiol levels were measured (A). Nonreducing SDS-PAGE was used to detect the extent of disulfide cross-linking (B), and the increase in cross-linking was determined and compared with native γSC (C). The bar graphs represent the mean ± SD of three independent experiments. *p < 0.05, **p < 0.01, ***p < 0.001, ****p < 0.0001, ns = not significant, M = molecular weight markers.

### Oxidation of Deamidated Proteins Further Increases AGE Levels in γSC

Glycation of oxidized and deamidated proteins resulted in a marked increase in AGE levels compared with glycation of only deamidated proteins (Figure 6A). The N14D (111.3%, *p* < 0.0001), N143D (150.1%, *p* < 0.0001) and TD (108.0%, *p* < 0.0001) showed significantly higher levels of CML. The double deamidated N14DN76D (32.5%, *p* < 0.0001), N14DN143D (57.0%, *p* < 0.0001) and N76DN143D (102.5%, *p* < 0.0001) also showed significant increases (Figure S5). The CML levels were highest in N143D, followed by N14D, TD and N76D143D. In general, the CML levels were nearly 27.7–267.2% higher in oxidized and deamidated γSC than in only deamidated γSC. The CEL levels were similarly elevated in oxidized deamidated γSC. N14D exhibited the highest levels (128.2% more than nonglycated, *p* < 0.0001). This was followed by N143D (Figure 6B). All three double-deamidated proteins showed approximately 50.8–72.5% CEL levels (*p* < 0.0001) (Figure S5). Unlike only deamidated proteins (Figure 3), in all deamidated and oxidized proteins, MG-H3 levels were significantly higher (*p* < 0.0001, Figures 6C and S5). The levels were 584.8–1190.6% higher than in native γSC.

**Figure 6:**
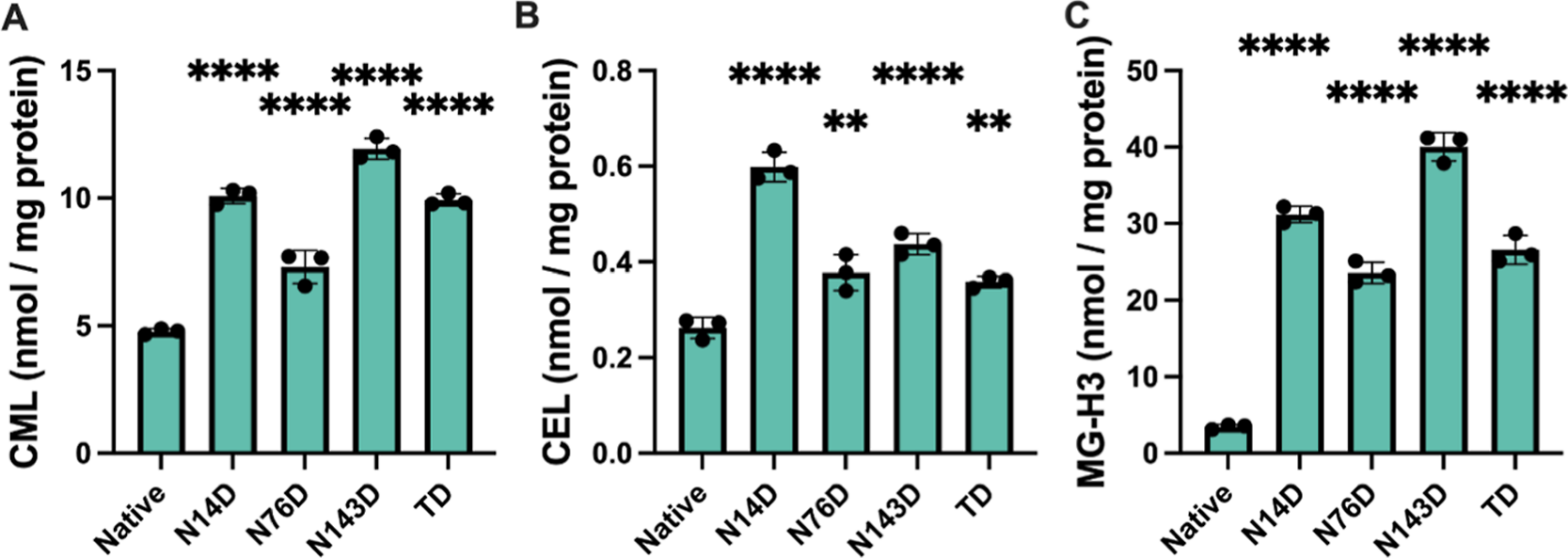
Oxidation increases the formation of non-cross-linking AGEs in deamidated γSC. Oxidation was performed as described in Figure 4. Native and mutant γSC were glycated and processed as in Figure 1. LC–MS/MS analysis of CML (A), CEL (B), and MG-H3 (C). The bar graphs represent the mean ± SD of three independent experiments. **p < 0.01, ****p < 0.0001, ns = not significant.

The cross-linking AGE and GOLD levels were significantly higher in all oxidized deamidated proteins except for N14D. The highest levels were observed with TD (990.8% higher, *p* < 0.0001), followed by N76DN143D (504.5% higher, *p* < 0.0001), and N14DN143D (471.3% higher, *p* < 0.0001) (Figures 7A and S6). Unlike GOLD, the MOLD levels were significantly higher only in TD (121.1% higher, *p* < 0.001) and N76DN43D (59.3% higher, *p* < 0.05) (Figures 7B and S5). Pentosidine levels were significantly higher in all deamidated and oxidized proteins, except N14DN76D. The highest levels were observed in N14D (362.4% higher, *p* < 0.001) and N14DN143D (428.0% higher, *p* < 0.0001) (Figures 7C and S6). It is conceivable that deamidation at N14 and N143 consistently promoted AGE formation in the oxidized deamidated proteins. This pattern was similar to what we observed in deamidated proteins alone.

**Figure 7:**
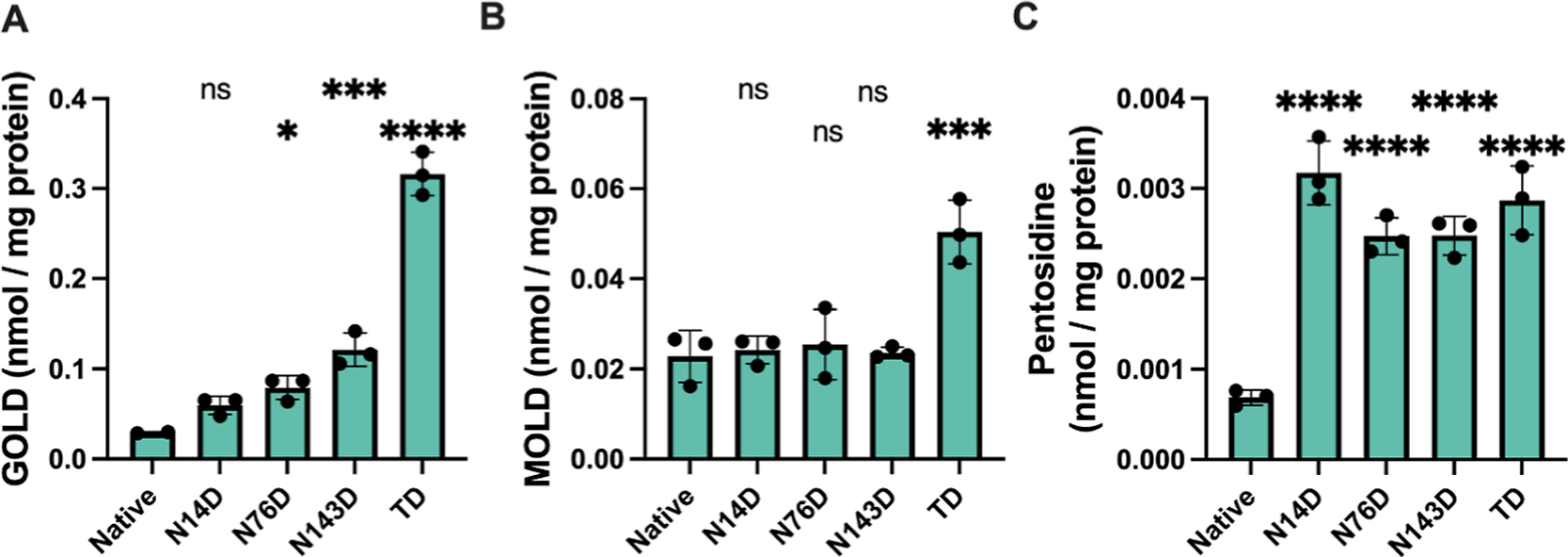
Oxidation increases the formation of cross-linking AGEs in deamidated γSC. Oxidation was performed as described in Figure 4. Native and mutant γSC were glycated and processed as in Figure 1. The proteins were dialyzed, acid hydrolyzed, and subjected to GOLD (A) MOLD (B) and pentosidine (C) measurements by LC–MS/MS. The bar graphs represent the mean ± SD of two or three independent experiments. *p < 0.05, ***p < 0.001, ****p < 0.0001, ns = not significant.

Despite the formation of cross-linking AGEs, surprisingly, intermolecular cross-linking did not increase in either glycated deamidated or glycated deamidated and oxidized proteins compared with their nonglycated counterparts (Figures S7 and S8). This suggests that glycation does not produce intermolecular cross-linking of γSC, but that cross-linking is likely to occur intramolecularly.

## Discussion

Deamidation in proteins is one of the major mechanisms of protein aggregation in the lens,^5,23,24,42^ and it begins very early in life.^6^ Deamidation introduces a negative charge at the site and increases the overall negative surface charge, as shown by zeta potential measurements (Figure S9). This may cause electrostatic repulsion between cysteine thiolates and proximal negatively charged deamidated residues,^5^ weakening local secondary structure, leading to partially unfolded regions, and promoting intermolecular disulfide formation. Subsequent oxidation (as performed in this study, and possibly in aging lenses) may lead to enhanced disulfide-mediated oligomerization, as observed here.

Deamidated crystallins are more prevalent in cataractous lenses than in noncataractous lenses.^24,43^ Deamidated crystallins are found at higher levels in the water-insoluble proteins compared to water-soluble proteins of cataractous lenses,^3,44,45^ indicating their role in protein insolubilization during cataract formation. Whether destabilization of the protein structure by deamidation and subsequent oxidation leading to disulfide cross-linking affects AGE formation has never been studied.

Our results showed that, in general, the formation of AGEs is higher in deamidated γSC than in native γSC. The AGEs formed in lysine residues (CML and CEL), as well as those formed between lysine–lysine residues (GOLD and MOLD) and lysine–arginine residues (pentosidine), were all elevated in deamidated γSC. This pattern also applied to deamidated and oxidized γSC. We compared the fold-change in total AGEs across all mutant variants relative to native γSC. The results suggested that most deamidated proteins showed higher levels, and that N14D, N143D and TD showed a consistent, nearly 100% increase (Figure S10). This indicates that deamidated proteins are more reactive in forming AGEs. The increase in reactive amines after deamidation, possibly arising from surface-exposed lysine residues, could increase AGE formation. In addition, the presence of acidic amino acid residues proximal to lysine residues has been shown to enhance glycation at those lysines.^46^ Therefore, deamidation of N14, which converts into an acidic residue (D14), could promote glycation of K15 in γSC. Deamidation may also alter the polarity and electrostatic properties of the surrounding microenvironment,^47^ and as a result, lysine residues proximal to newly formed aspartic acid residues may become more susceptible to glycation. However, additional studies are needed to confirm site-specific AGE formation following deamidation.

Whether deamidation exposed additional lysine residues and thereby enabled higher AGE formation was investigated. Results showed that deamidation increases the surface-exposed lysine residues (Figure S4). A previous hydrogen–deuterium exchange NMR study showed that deamidation at N14D and N76D increases the exchange rates of lysine residues, suggesting that those residues become more solvent-exposed.^23^ Therefore, it is likely that lysine residues in deamidated proteins are more surface-exposed, making them available for glycation and thereby contributing to increased AGE formation. However, our results showed no correlation between surface-exposed lysine residues and CML formation. Therefore, glycation itself might have caused subtle conformational changes in deamidated γSC, further exposing lysine residues and promoting CML formation. Another possibility is that after deamidation, subtle structural changes may have brought cysteine residues into proximity with lysine residues, which may have resulted in cysteine-promoted CML formation, analogous to observations in a previous study.^28^ These processes do not appear to occur uniformly across all deamidated proteins we tested. For example, N14DN76D showed a similar increase in surface-exposed lysine residues, as other deamidated variants did, but had lower CML levels. Thus, exposure of additional lysine residues by deamidation is not the sole determinant of AGE formation, and other factors that occur during glycation seem to dictate the eventual formation of AGEs.

Protein oxidation in the lens begins early in life, and the amount of insoluble protein and fluorescence attributable to AGEs increases exponentially after the age of 40 ^48,49^ suggesting that cumulative oxidative damage predisposes lens proteins to AGE-mediated cross-linking and insolubilization. However, whether proteins brought together by thiol oxidation become more susceptible to AGE-mediated cross-linking has never been studied. The result suggests that, after oxidation followed by glycation, native γSC showed ∼150% increase in CML compared to glycation alone. The total AGEs, which include CML, CEL, MG-H3, GOLD, MOLD, and pentosidine, are nearly twice the amount observed with only deamidation in native γSC (Figure S10 and Table S1). We also assessed MG-H1 levels, and the trends are largely similar to those of MG-H3 in both deamidated and deamidated-oxidized γSC (Figure S11), suggesting overall comparable reactivity in hydroimidazolone formation. Deamidation of γSC caused only minor alterations in its secondary and tertiary structures, as evidenced by CD and fluorescence studies (Figure 2). However, previous studies on γSC have shown that deamidation increases disulfide bond formation and causes structural alterations.^5,24^ Therefore, we hypothesized that oxidation occurring after deamidation would have a greater impact on AGE formation than deamidation alone. Our results support this possibility; we observed higher levels of AGEs in deamidated and oxidized proteins than in deamidated proteins alone.

The effect of deamidation on AGE formation might not be limited to γSC. Other proteins in the lens could also be impacted. α-Crystallin is the most abundant protein in the lens, and its deamidation has been well documented.^20,21^ It has been shown that deamidation of α-crystallin alters its oligomeric structure and its interactions with other crystallins.^50^ Deamidation also reduces α-crystallin’s chaperone activity.^42^ Deamidated in αAC binds to lens fiber cell plasma membranes more than the native protein.^51^ Deamidated α-crystallin may become more susceptible to AGE modification, similar to γSC, and could show increased abnormal activities mentioned above. Similarly, the other major class of proteins in the lens, β-crystallins, which have been shown to be deamidated in human lenses,^16,22^ might also become susceptible to AGE modifications. If so, this could significantly contribute to lens aging and cataract development. These possibilities should be further explored.

Deamidation-promoted AGE formation may be relevant in other diseases as well. In Alzheimer’s disease, deamidated forms of amyloid and tau accumulate in plaques and neurofibrillary tangles.^52^ Deamidated α-synuclein forms more aggregation-prone species in Parkinson’s disease,^53^ and deamidated SOD1 ^54^ and TDP-43 ^55^ have been observed in amyotrophic lateral sclerosis. In diabetes, many proteins, including serum albumin and skin collagen undergo deamidation.^56–58^ These deamidated proteins may become more susceptible to AGE modification. If deamidated proteins undergo further oxidation, AGE formation may accelerate, potentially hastening disease progression.

It is well-known that glycation and oxidation are interconnected mechanisms of protein aging. Oxidation of sugars and ascorbate produces highly reactive glycation initiators^59^ , which can generate reactive aldehydes and ketones that accelerate AGE formation in proteins. In addition to the established relationship between glycation and oxidation, the findings in this study uncover deamidation as an additional mechanism that can influence AGE formation. Thus, the combined effect of the three PTMs might be significant for protein aging. In this context, some highly AGE-modified proteins found in aged individuals (for example, skin collagen I) might also be relatively more deamidated and/or oxidized than other proteins that are less modified by AGEs, and this needs to be investigated.

## Conclusions

This study demonstrates that deamidation markedly increases the susceptibility of γSC to AGE formation, particularly at lysine residues, resulting in elevated levels of both non-cross-linking and cross-linking AGEs. In contrast, arginine-derived AGEs are largely unaffected. Deamidation at specific sites, especially N14D and N143D, plays a critical role in enhancing glycation. Oxidation further exacerbates these effects by promoting cross-linking and overall AGE accumulation, with the TD variant showing the highest. Oxidation also increases MG-H3 levels in deamidated γSC upon glycation. Collectively, these findings reveal an interplay among deamidation, oxidation, and glycation that drives protein cross-linking and aggregation, thereby contributing to lens aging.

## Supplementary Material



## Notes

Attached normalized fluorescence spectra of γSC and deamidated variants; LC–MS/MS data for AGEs of deamidated and native γSC before and after oxidation. TNBS assay for the determination of surface-exposed lysine residues. SDS-PAGE gels to compare intermolecular protein cross-linking. Zeta potential of native and deamidated γSC. Comparative analysis of total AGEs. Table showing levels of all AGEs (PDF)

## Abbreviations

PTMs: post-translational modifications
AGEs: advanced glycation endproducts
γSC: γS-Crystallin
MGO: methylglyoxal
αAC: αA-Crystallin
αBC: αB-Crystallin
GSH: reduced glutathione
GSSG: oxidized glutathione
CML: *N* ^ε^-carboxymethyllysine
CEL: *N* ^ε^-carboxyethyllysine
GOLD: glyoxal-lysine dimer
MOLD: methylglyoxal-lysine dimer
MG-H3: methylglyoxal-derived hydroimidazolone
