# Supplementary material for: Deamidation Promotes AGE-Modifications in Human Lens γS-Crystallin
Source: Biochemistry. 2026 Apr 7;65(12):1957–65. doi: 10.1021/acs.biochem.6c00087 (PMC13276836; doi:10.1021/acs.biochem.6c00087)
Supplement: Supplementary file 1 [file bi6c00087_si_001.pdf]

Supporting Information

Deamidation Promotes AGE-Modifications in Human Lens γS-crystallin

Sudipta Panja[1] and Ram H. Nagaraj[1,*]

[1]University of Colorado Anschutz Department of Ophthalmology, Aurora, CO 80045, USA

*** Correspondence:**

Ram H. Nagaraj, Ph.D. at: ram.nagaraj@cuanschutz.edu

Figure S1

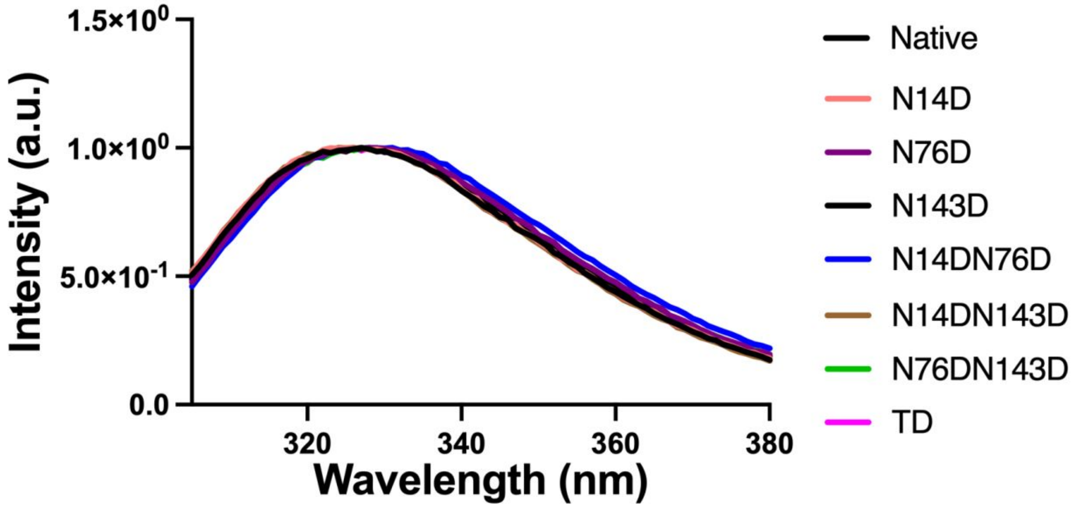

Figure S1. Normalized fluorescence spectra (from Figure 2D) show a slight blue shift of the emission maximum, indicating a more solvent-exposed tertiary structure after deamidation.

Figure S2

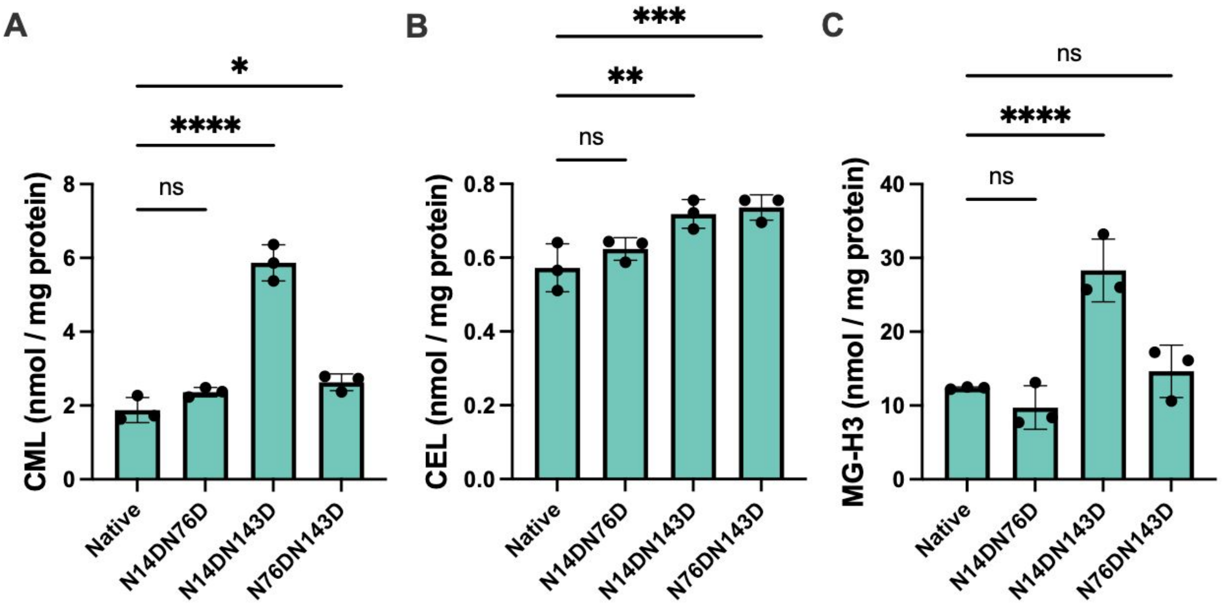

Figure S2. Native and deamidated γSC were incubated with 25 mM D-glucose, 2 mM ascorbic acid, and 250 μM MGO for five days at 37°C, as shown in Figure 3. The proteins were dialyzed, acid hydrolyzed and analyzed for CML (A) and CEL (B) MG-H3 (C) by LC-MS/MS. The bar graphs represent the mean ± SD of three independent experiments. *$p<0.05$, **$p<0.01$, ***$p<0.001$, ****$p<0.0001$, ns=not significant.

Figure S3

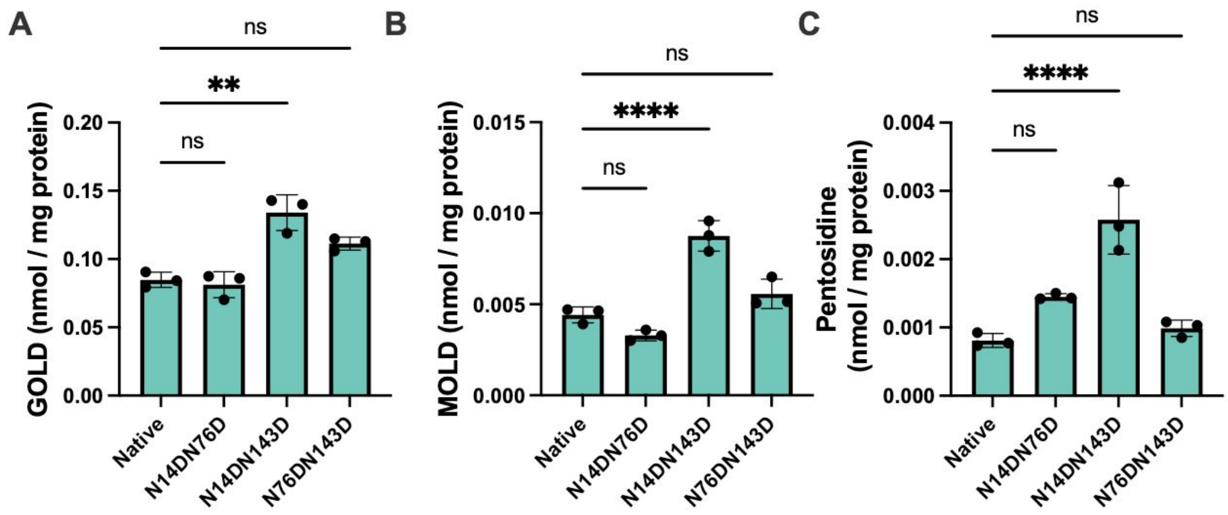

Figure S3. Native and deamidated γSC were glycated and processed as in Figure 3, and subjected to GOLD (A) and MOLD (B) and pentosidine (C) analysis by LC-MS/MS. The bar graphs represent the mean ± SD of three independent experiments. **p<0.01, ****p<0.0001, ns=not significant.

Figure S4

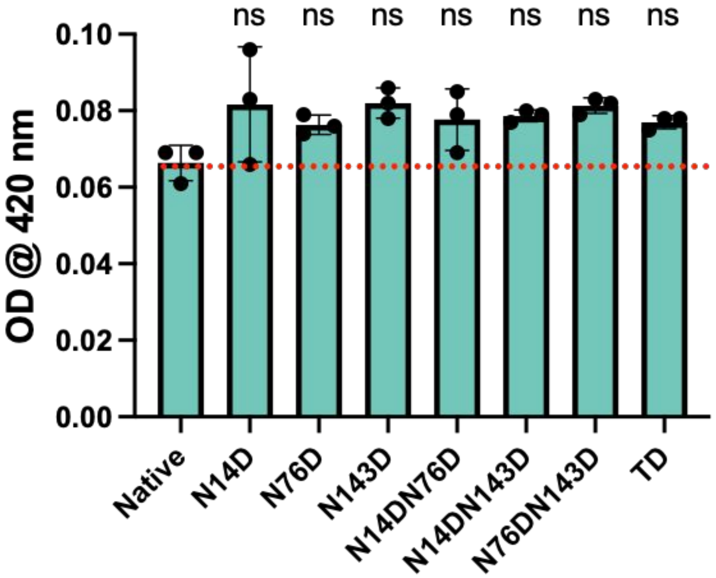

Figure S4. Surface-exposed lysine residues, determined by the TNBS assay. The red dotted line indicates the level in the native protein. ns=not significant.

Figure S5

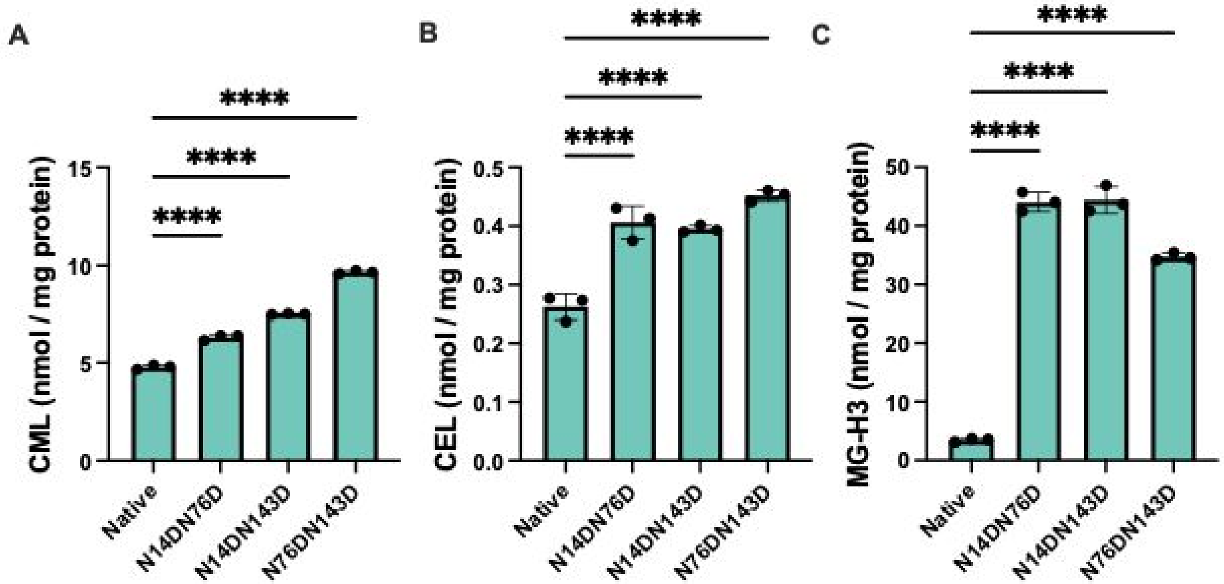

Figure S5. Oxidation was performed as described in Figure 5. The proteins were glycated and processed as shown in Figure 2. The bar graphs represent the mean ± SD of three independent experiments. ****$p<0.0001$.

Figure S6

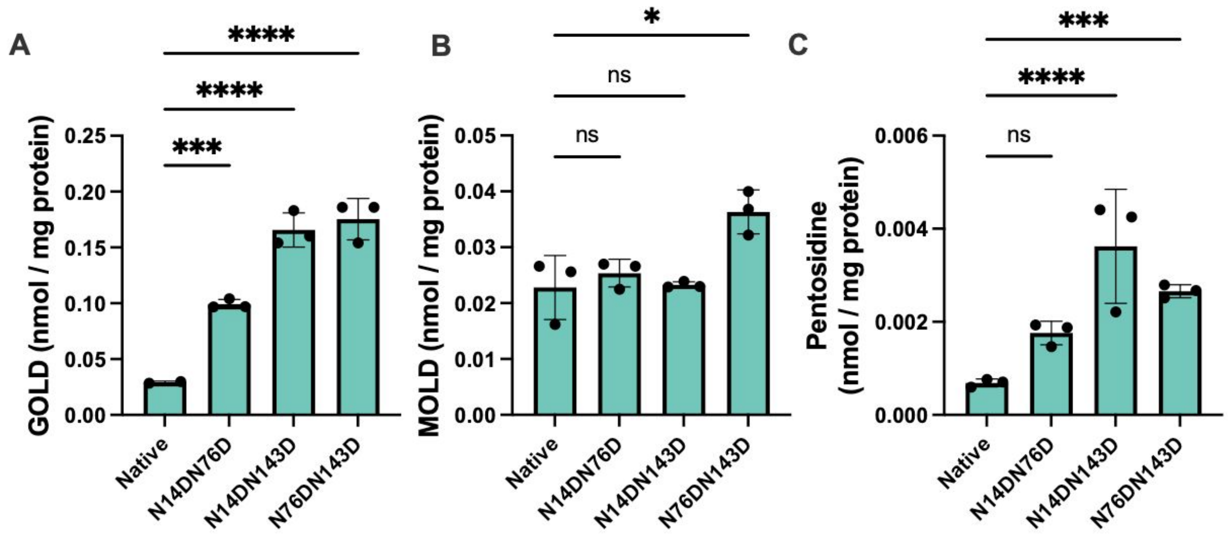

Figure S6. Oxidation was performed as described in Figure 5. Native and γSC mutants were glycated and processed as in Figure 2. The proteins were dialyzed, acid hydrolyzed, and subjected to GOLD (A) MOLD (B) and pentosidine (C) measurements by LC-MS/MS. The bar graphs represent the mean ± SD of two or three independent experiments. *p<0.05, ***p<0.001, ****p<0.0001, ns=not significant.

Figure S7

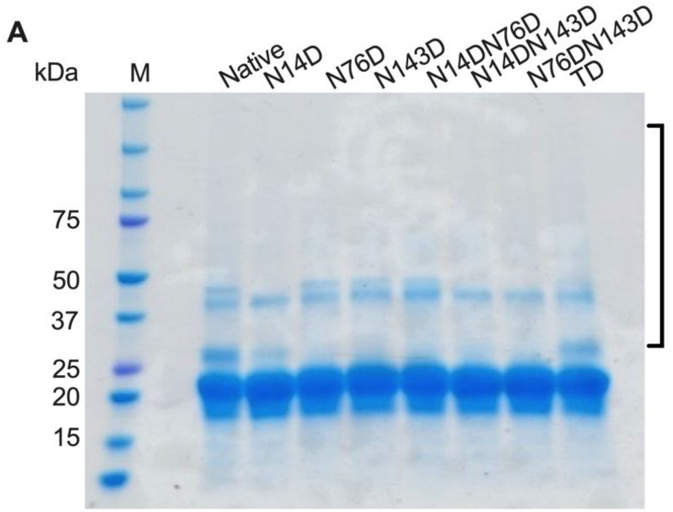

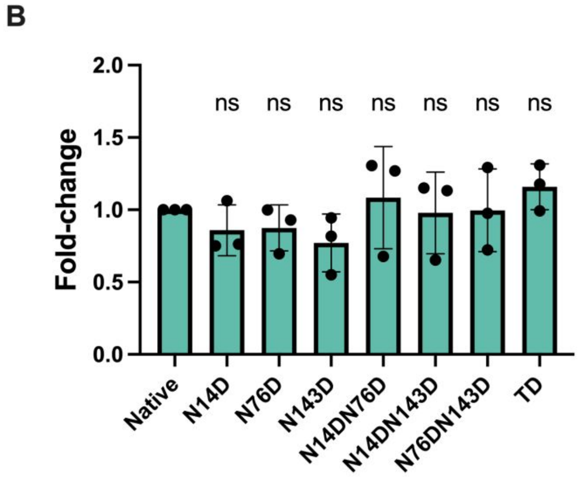

Figure S7. Native and deamidated γSC were glycated as described in Figure S3. The protein samples were dialyzed and subsequently analyzed by electrophoresis on a 4–20% gradient SDS-PAGE gel. The bar graph (densitometry plot for the regions in brackets) shows the mean ± SD of three independent experiments (B). ns=not significant. M=molecular weight markers.

Figure S8

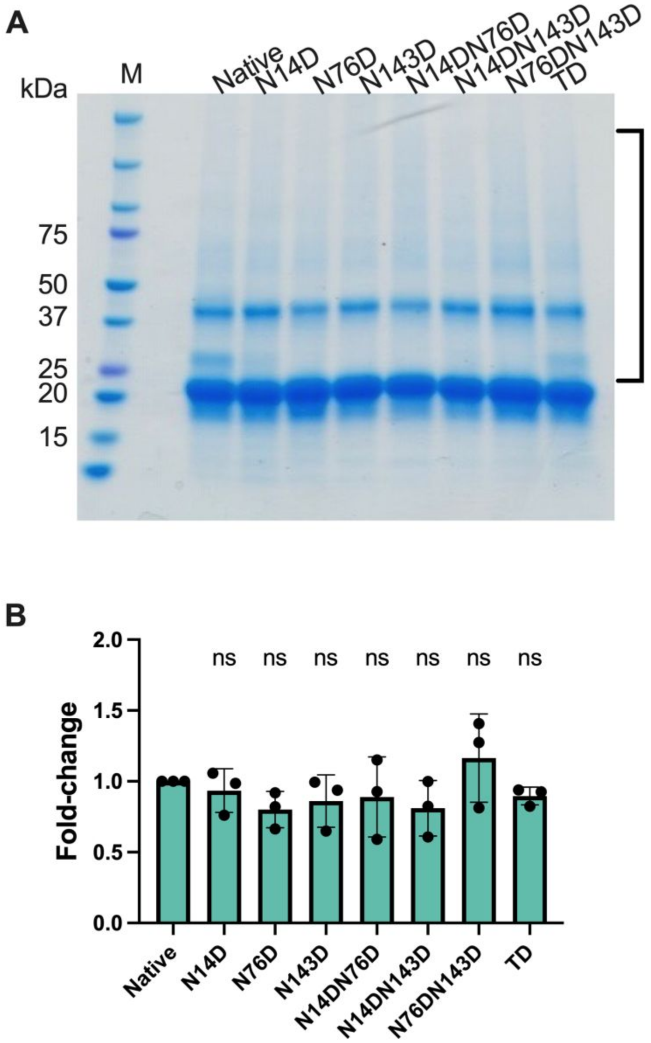

Figure S8. After oxidizing with 2 mM GSSG, native and deamidated γSC were incubated with the glycating mixture as described in Figure S3 and analyzed by electrophoresis on a 4–20% gradient SDS-PAGE gel (A). The bar graph (densitometry plot for the regions in brackets) is the mean ± SD of three independent experiments (B). ns=not significant. M=molecular weight markers.

Figure S9

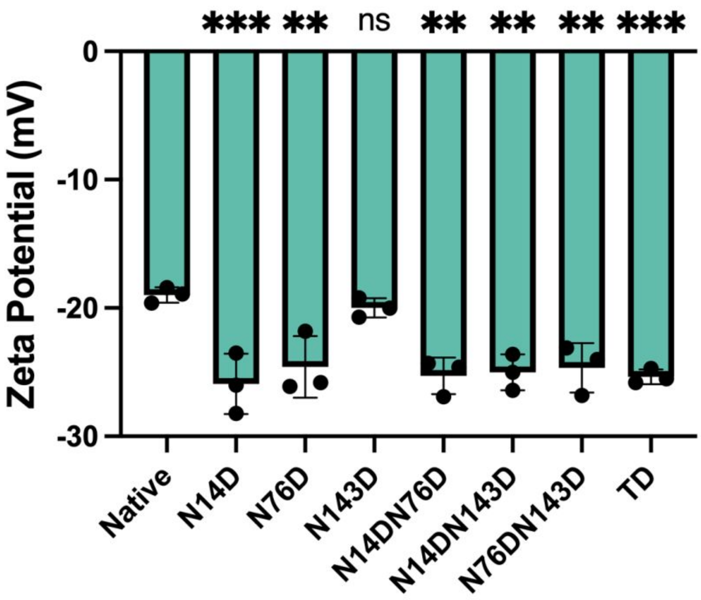

Figure S9. The zeta potential of native and deamidated γSC. **p<0.01, ***p<0.001, ns=not significant.

Figure S10

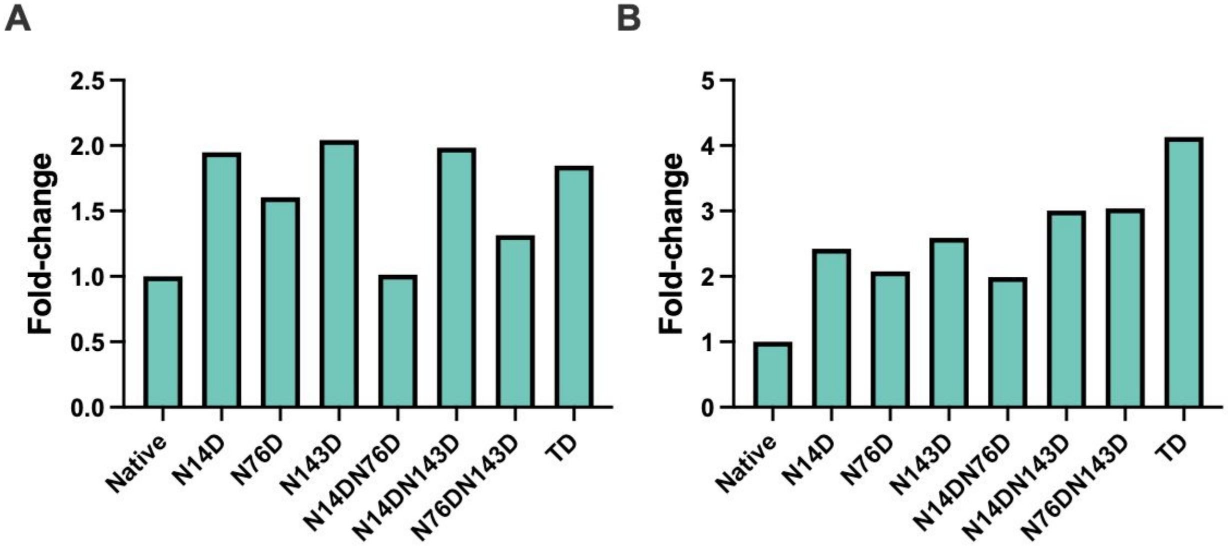

Figure S10. Each AGE was normalized relative to native γSC and the aggregated AGE levels were expressed as fold-change over non-glycated γSC. For calculation, the mean of 2-3 independent values was used. A=deamidated and glycated and B=deamidated, oxidized and glycated.

Figure S11

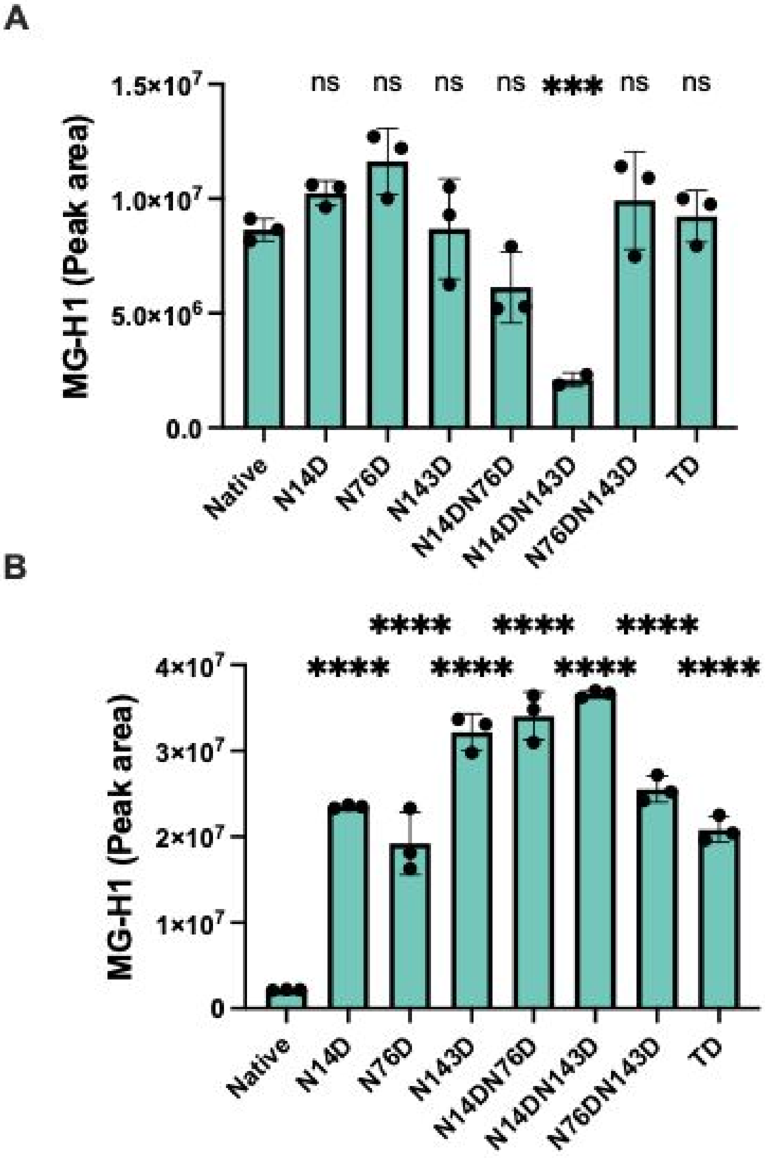

Figure S11. Deamidated (A) or deamidated and oxidized (B) γSC were incubated with 25 mM D-glucose, 2 mM ascorbic acid, and 250 μM MGO for five days at 37°C. MG-H1 levels were measured by LC-MS/MS. ***p<0.001, ****p<0.0001, ns=not significant.

Table S1.

A

	**CML (nmol/mg Protein)**	**CEL (nmol/mg Protein)**	**MG-H3 (nmol/mg Protein)**	**GOLD (nmol/mg Protein)**	**MOLD (nmol/mg Protein)**	**Pentosidine (nmol/mg Protein)**
**Native**	1.88±0.34	0.58±0.07	12.36±0.15	0.08±0.01	0.004±0.001	0.001±0.001
**N14D**	4.63±0.13	0.56±0.05	15.26±0.40	0.14±0.02	0.012±0.001	0.002±0.001
**N76D**	4.12±0.32	0.33±0.03	16.7±2.10	0.17±0.02	0.007±0.001	0.001±0.001
**N143D**	5.52±0.38	0.96±0.04	13.66±2.72	0.13±0.01	0.009±0.001	0.002±0.001
**N14DN76D**	2.36±0.13	0.62±0.03	9.73±2.94	0.08±0.01	0.004±0.001	0.001±0.001
**N14DN143D**	5.87±0.49	0.72±0.04	28.30±4.24	0.13±0.01	0.009±0.001	0.002±0.001
**N76DN143D**	2.63±0.23	0.74±0.03	14.63±3.53	0.11±0.01	0.006±0.001	0.001±0.001
**TD**	4.44±0.17	0.74±0.01	13.07±1.33	0.18±0.02	0.007±0.001	0.002±0.001

B

	**CML (nmol/mg Protein)**	**CEL (nmol/mg Protein)**	**MG-H3 (nmol/mg Protein)**	**GOLD (nmol/mg Protein)**	**MOLD (nmol/mg Protein)**	**Pentosidine (nmol/mg Protein)**
**Native**	4.77±0.12	0.26±0.02	3.44±0.31	0.03±0.01	0.02±0.01	0.001±0.001
**N14D**	10.08±0.30	0.60±0.03	31.20±1.05	0.06±0.01	0.02±0.01	0.003±0.001
**N76D**	7.30±0.64	0.38±0.03	23.57±1.39	0.08±0.01	0.03±0.01	0.002±0.001
**N143D**	11.93±0.42	0.44±0.02	40.03±1.85	0.12±0.02	0.02±0.01	0.002±0.001
**N14DN76D**	6.32±0.14	0.41±0.03	44.07±1.60	0.10±0.01	0.03±0.01	0.002±0.001
**N14DN143D**	7.49±0.03	0.40±0.01	44.40±2.23	0.17±0.02	0.02±0.01	0.004±0.001
**N76DN143D**	9.66±0.08	0.45±0.01	34.70±0.62	0.18±0.02	0.04±0.01	0.003±0.001
**TD**	9.93±0.24	0.36±0.01	26.57±1.89	0.32±0.02	0.05±0.01	0.003±0.001

Table S1. AGE levels were determined by LC-MS/MS after incubation of proteins with 25 mM D-glucose, 2 mM ascorbic acid, and 250 µM MGO for five days at 37°C (A). Oxidation was performed using GSSG (2 mM); thereafter, native γSC and all mutants were glycated and analyzed for AGEs by LC-MS/MS (B).
